# Association between vitamin D level and cataract: A systematic review and meta-analysis

**DOI:** 10.1007/s00417-024-06592-w

**Published:** 2024-08-23

**Authors:** Zhenzhen Jue, Ziming Xu, Vincent L. Yuen, Odessa Dik San Chan, Jason C. Yam

**Affiliations:** 1 Department of Ophthalmology and Visual Sciences, The Chinese University of Hong Kong, 147K Argyle Street, Kowloon, Hong Kong SAR, China; 2 Hong Kong Eye Hospital, Kowloon, Hong Kong SAR, China; 3 Department of Ophthalmology, Hong Kong Children’s Hospital, Hong Kong SAR, China; 4 Hong Kong Hub of Paediatric Excellence, The Chinese University of Hong Kong, Hong Kong SAR, China

**Keywords:** Cataract, Vitamin D, Serum vitamin D level, Types of cataract

## Abstract

**Purpose:**

The association between serum vitamin D level and cataract remains controversial. This study aims to evaluate the association between vitamin D level and cataract.

**Methods:**

In this study, articles in the PubMed, Web of Science, and EMBASE databases were searched up to 30 August 2023 and 626 articles were screened. Four studies involving a total of 10,928 subjects with cataract and 10,117 control subjects met the inclusion criteria.

**Results:**

Decreased serum vitamin D level was associated with higher incidence of cataract (P = 0.047; MD: -4.87; 95%CI: [-9.67, -0.07]). In the subgroup analysis by sex, a significant association was found between serum vitamin D level and cataract in both male (P = 0.01, MD: -2.15,95%CI: [-3.83, -0.46]) and female (P < 0.01; MD: -6.67,95%CI: [-8.20, -5.14]).In the subgroup analysis by the types of cataract, significant association was found between serum vitamin D level and nuclear (P < 0.01; MD: -10.48; 95%CI: [-12.72, -8.24]) and posterior subcapsular cataract (P = 0.02; MD: -6.05; 95%CI: [-11.30, -0.80]) but not in cortical cataract (P = 0.14; MD: -6.74; 95%CI: [-15.70, 2.22]).

**Conclusion:**

This meta-analysis revealed potential association between serum vitamin D level and cataract, more significant in female, and the subtypes of nuclear and posterior subcapsular cataract.

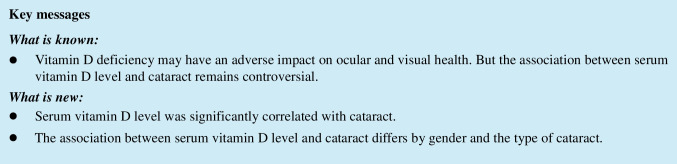

**Supplementary Information:**

The online version contains supplementary material available at 10.1007/s00417-024-06592-w.

## Introduction

Cataract, opacification of the lens, is the main cause of blindness and visual impairment in middle-income and low-income countries [1, 2]. Studies have shown that of the 36 million people who are blind worldwide, about 1/3 of them are due to cataract and among the population of 216.6 million people with moderate or severe visual impairment, 52.6 million of them are attributed to cataract [3]. It is a complex multifactorial disease influenced by various genetic and environmental factors [4, 5]. Vitamin D plays various roles in preserving human health, encompassing the regulation of gene expression, anti-inflammatory, and anti-apoptotic properties [6]. A number of studies have shown that vitamin D deficiency may have an adverse impact on ocular and visual health, such as age-related macular degeneration, glaucoma and diabetic retinopathy [7–13].

Although clinical studies of vitamins and cataract have been the subject of frequent inquiry [14, 15], compared to other types of vitamins, there is still controversy about the association between vitamin D and cataract. Aidenloo reported that serum 25(OH) D level were inversely associated with cataract in women, and no such association has been found in men [16]. In contrast, Jee et, al concluded that serum vitamin D was associated with cataract in men and not in women [17]. There is also controversy regarding different types of cataract. Park suggested negative association between serum 25(OH) D level and the risk of nuclear cataract [18], while Rao's study found no significant association between the two [19].

However, so far, there is no systematic review on the association between vitamin D and cataract, and in order to advance knowledge based on the available evidence, we conducted a systematic review and meta-analysis with the aim of assessing the association between serum vitamin D level and cataract.

## Methods

We conducted and reported this systematic review following the latest PRISMA guidelines [20]. The study protocol was registered on PROSPERO under the identification number CRD42023467576.

### Search strategy

We searched three databases: PubMed, Embase, and Web of Science. These were published in English from the inception of each database to 30 August 2023. The search strategy consisted of two main elements connected by the AND operator: (1) cataract; and (2) vitamin D. For each of these core components, we identified controlled vocabulary (specifically, Medical Subject Headings [MeSH] terms) as well as relevant keywords. Complete information regarding all search terms can be found in the supplemental file.

### Inclusion criteria and study selection

All studies had to meet the following inclusion criteria: (1) Two or more comparison groups (cataract and control groups) were included; (2) Studies with an outcome of a laboratory assessment of serum or plasma vitamin D level; (3) The study was published in English; (4) The full text of the article was accessible; and (5) The subjects were human. Animal studies, case reports, reviews, abstracts and editorials were excluded.

The articles were imported into a reference management software (Covidence; Veritas Health Innovation Ltd) for the purpose of study selection. Two reviewers (ZZJ and ZMX) independently assessed the titles and abstracts of the studies for eligibility. In cases of disagreement, a third reviewer (VLY) resolved the discrepancies. All studies identified as potentially eligible based on the title and abstract screening underwent a comprehensive full-text review by two independent reviewers (ZZJ and ODSC) using the same predetermined criteria. Any discrepancies in eligibility ratings were addressed through discussion or involvement of the third reviewer (VLY).

#### Data extraction and risk of bias assessment

From each article, the following data were collected and reviewed independently by two reviewers (ZZJ and ZMX): first author, publication year, country/region, age, sample size, male and female size, study design, type of cataract and considered vitamin D. The data extracted by the two reviewers were consistent. The risk of bias in case–control studies and cross-sectional study were assessed using the Newcastle–Ottawa Scale, with ratings categorized as high (≥ 8 stars), moderate (6–7 stars), or low (< 6 stars) [21].

### Statistical analysis

For each study included in the analysis, we calculated the mean difference (MD) in vitamin D level between the cataract and control groups, along with their corresponding 95% confidence interval (CI). To assess the heterogeneity among the pooled studies, we utilized both the χ^2^-based Q statistic and I^2^ metrics. In cases where heterogeneity was detected (p < 0.05 or I^2^ > 50%), a random-effects model was used; otherwise, a fixed-effects model was used. Meanwhile, we conducted subgroup analysis to assess the potential heterogeneity by gender and different types of cataract. For studies that only reported the means and standard deviations of individual subgroups, we followed the recommended approach by the Cochrane system to combine the mean and standard deviation of the two reported subgroups into a single group [22]. Furthermore, we conducted a sensitivity analysis using the leave-one-out strategy to examine the stability of the results. This involved systematically excluding individual studies from the analysis and assessing if their omission significantly affected the overall findings. For assessing publication bias in the included articles, we used the Egger's test. Statistical significance was defined as a p-value less than 0.05. All statistical analyses were performed using R (version 4.2.3).

## Results

### Search results and study characteristics

**Fig. 1:**
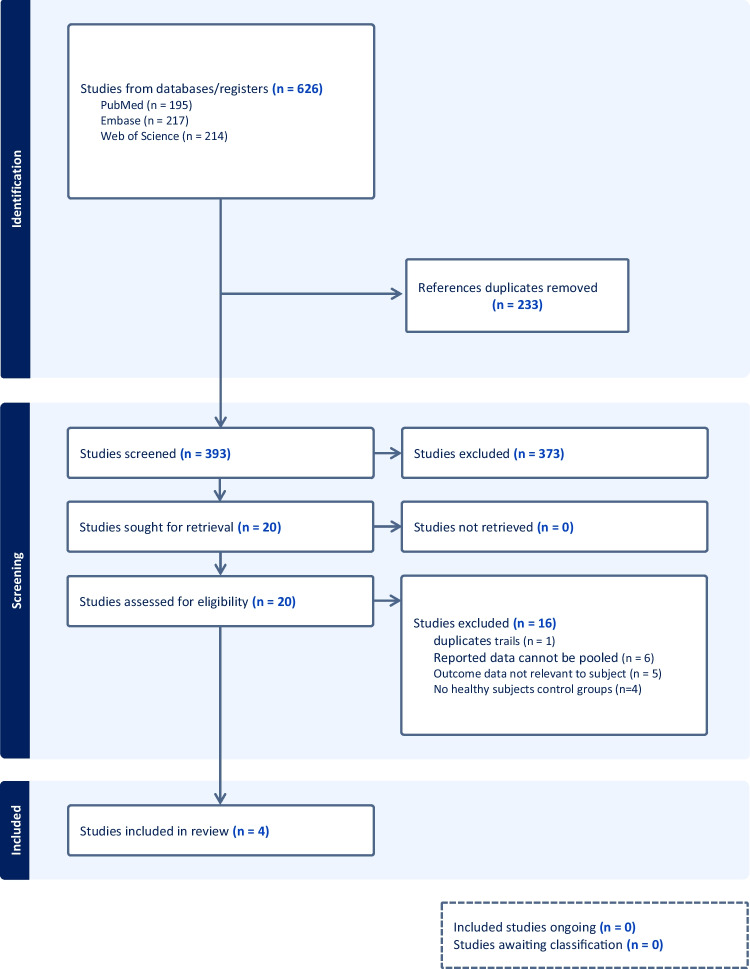
PRISMA Flow Diagram

During the initial search of the three databases, a total of 626 studies were identified. After removing duplicate publications, 393 articles were screened for eligibility. Of the 20 studies eligible for full-text review, 4 met the inclusion criteria (Fig. 1 presented a flowchart that illustrated the process of conducting the article search in the study). Table 1 summarizes the study characteristics, designs, and findings. The final analysis included four studies, consisting of three case–control studies [16, 23, 24] and one cross-sectional studies [17]. Among them, two studies analyzed different types of cataract [16, 23], and three studies conducted analyses based on different genders [16, 17, 24]. The results of the quality assessment of the included studies are shown in Table 2. The quality score of these studies ranged from 6 to 7.

**Table 1:** Characteristics of included studies of vitamin D and cataract

Author, Year	Country	Study design	Different cataract type	Assay for vitamin D	Cataract group				Control group			
					No	Male	Age	Vitamin D, ng/ml	No	Female	Age	Vitamin D, ng/ml
Abdellah 2019 [23]	Egypt	Case–control study	Yes	Serum,25(OH)D3; CMIA	325	155	63.1 ± 6.3	7.6 ± 5.5	385	180	62.9 ± 6.7	18.5 ± 9.6
Aidenloo 2022 [16]	Iran	Case–control study	Yes	Serum,25(OH)D3; CMIA	1241	636	64.7 ± 8.5	30.3 ± 9.9	200	100	62.6 ± 8.0	34.5 ± 8.5
Jee 2015 [17]	Korea	Cross-sectional study	NO	Serum,25(OH)D3; Radioimmunoassay	9325	Conflict	63.6 ± 19.3	19.1 ± 9.7	9479	Conflict	49.5 ± 9.7	18.4 ± 9.7
Öktem 2021 [24]	Turkey	Case–control study	NO	Serum,25(OH)D3 CMIA	37	18	48.1 ± 8.5	15.6 ± 8.4	53	27	49.3 ± 7.8	20.8 ± 7.1

*CMIA*: chemiluminescent microparticle immunoassay

**Table 2:** Quality of included studies

Author, Year	Selection				comparability	Exposure			Overall score	Quality
	Case definition adequate	Representativeness of the cases	Selection of controls	Definition Of controls	Comparability of cases and controls on the bias of the design or analysis	Ascertainment of exposure	Same method of ascertainment for cases and controls	Non-response rate		
Abdellah 2019 [23]	1	1	0	1	1	1	1	0	6	Medium
Aidenloo 2022 [16]	1	1	1	1	1	1	1	0	7	Medium
Jee 2015 [17]	1	1	1	NA	1	1	1	0	6	Medium
Öktem 2021 [24]	1	1	0	1	1	1	1	0	6	Medium

*NA*: not applicable

### Association between blood 25(OH)D concentration and cataract

**Fig. 2:**
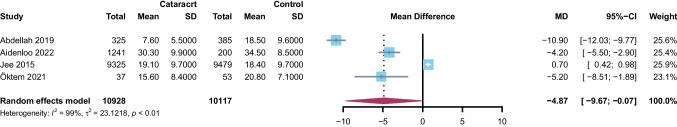
Forest plot of the mean differences in the serum 25(OH)D level between cataract and control groups

In all, the 4 studies had a total of 10,928 patients in the cataract group and 10,117 patients in the control group. The blood 25(OH)D level in the cataract group was lower than control group (P = 0.047, MD: -4.87,95%CI: [-9.67, -0.07]; Fig. 2), and there was significant heterogeneity among studies (I^2^ = 99%, *p* < 0.01).

### Subgroup analysis

#### Sex

**Fig. 3:**
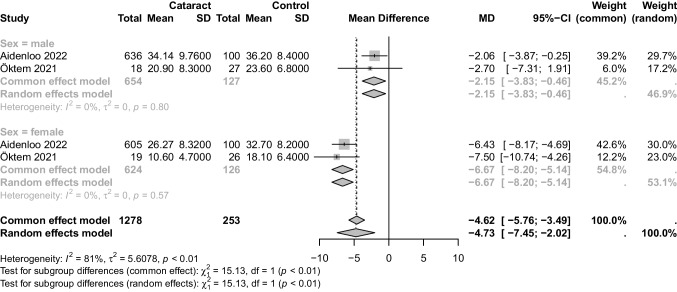
Forest plot of subgroup analysis of the associations between serum 25(OH)D level and cataract in different gender groups

There were three studies that investigated the relationship between vitamin D and cataract in male and female subgroups. However, due to conflicting participant numbers of male and female in one of the studies, that particular study was excluded from the analysis [17]. As a result, a total of two studies were included in the analysis. Significant association between the serum 25(OH)D level and cataract was found in male (P = 0.01, MD: -2.15,95%CI: [-3.83, -0.46], I^2^ = 0; Fig. 3) and female(*P* < 0.01, MD: -6.67,95%CI: [-8.20, -5.14], I^2^ = 0; Fig. 3).

### Different types of cataract

**Fig. 4:**
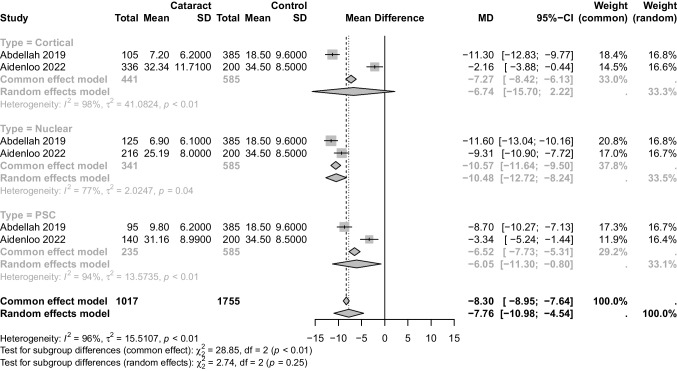
Forest plot of subgroup analysis of the association between serum 25(OH)D level and different types of cataract

Significant associations with serum 25(OH)D level were found in nuclear cataract (P < 0.01, MD: -10.48,95%CI: [-12.72, -8.24], I^2^ = 77%; Fig. 4) and posterior subcapsular cataract (P = 0.02, MD: -6.05,95%CI: [-11.30, -0.80], I^2^ = 94%; Fig. 4) but not in cortical cataract (*P* = 0.14, MD: -6.74,95%CI: [-15.70, 2.22], I^2^ = 98%; Fig. 4) and control group,

### Risk of bias

**Fig. 5:**
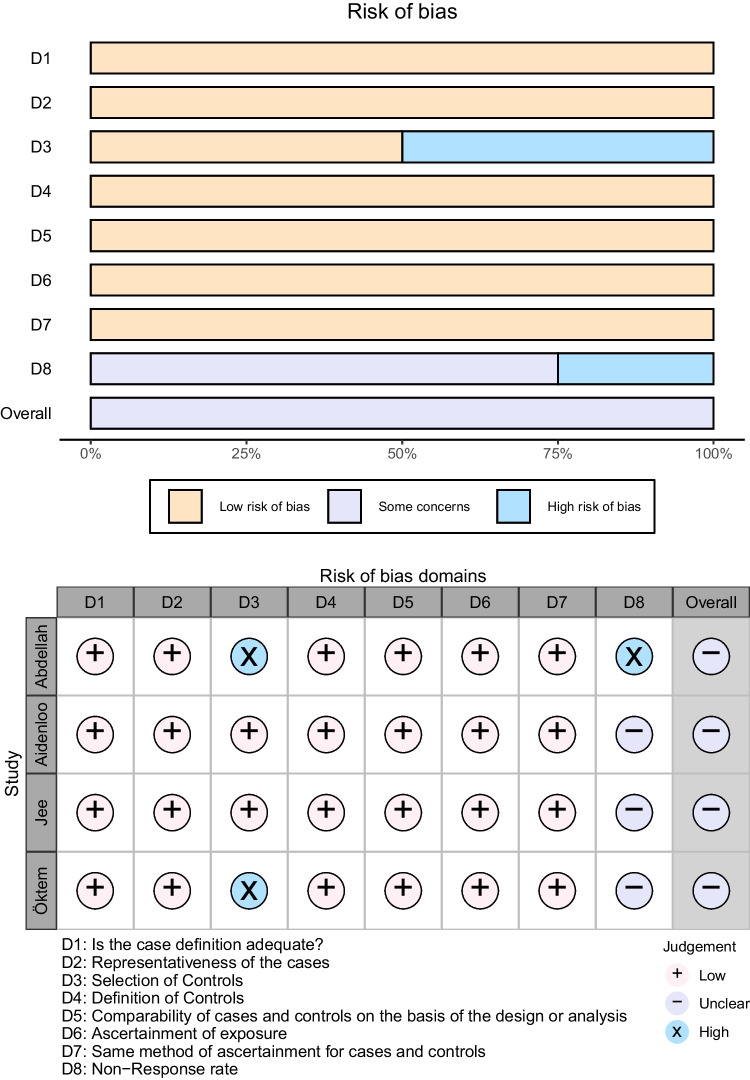
Risk of bias of all included studies

We assessed risk of bias by using the quality of the studies. All four articles were rated as having some concerns. Domain level and overall risk of bias for studies are presented in Fig. 5. There was no significant difference in publication bias (*t* = -1.66, P = 0.24). Moreover, we only included studies published in English, so there was a high risk of publication language bias.

### Sensitivity analysis

**Fig. 6:**
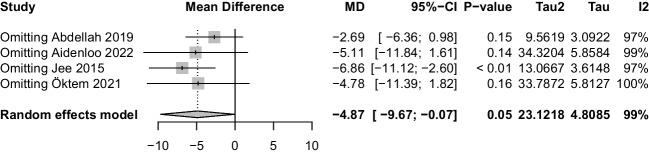
Forest plot of sensitivity analysis

We conducted sensitivity analysis for these studies, the result showed that one study influenced the meta-analysis results. (Fig. 6).

### Heterogeneity

#### Statistical heterogeneity

Three of the included studies [16, 23, 24] reported serum vitamin D level using mean and standard deviation (SD), while one study [17] used mean and standard error (SE). Aidenloo [16] conducted analyses on the relationship between serum vitamin D level and cataract separately for different genders and different types of cataract, without analyzing the entire population as a whole. Therefore, there is a certain degree of heterogeneity statistically.

### Clinical heterogeneity

The four included studies recruited patients from different age groups. Abdellah [23] and Aidenloo [16] included cataract patients with an average age of around 60 years. Jee [17] included participants from Korea National Health and Nutrition Examination Survey (KNHANES), with the cataract group having a mean age of 63.6 and the control group having a mean age of 49.5. Additionally, Öktem included a population of young adult cataract patients aged below 60 years. Furthermore, one study was conducted in Africa [23], while the remaining three studies were conducted in Asia [16, 17, 24].

### Methodological heterogeneity

The four included studies all measured serum 25-hydroxyvitamin D level. However, there were differences in the measurement methods and study designs among the studies. Abdellah, Aidenloo, and Öktem utilized chemiluminescent microparticle immunoassay, [16, 23, 24] while Jee used radioimmunoassay for measuring vitamin D level [17]. Furthermore, Abdellah, Aidenloo, and Öktem conducted case–control studies [16, 23, 24], while Jee conducted a population-based cross-sectional study [17].

## Discussion

This is the first meta-analysis of the association between serum vitamin D level and cataract. In our study, a total of 4 studies were included, which included 10,928 participants in the cataract group and 10,117 participants in the control group. Our study suggested that a negative association between serum vitamin D level and cataract, supporting previous studies that reported similar results [23–25]. There may be various underlying mechanisms. Vitamin D has been shown to possess antioxidant properties, which may protect against oxidative stress and cellular damage in the lens of the eye [26, 27]. Additionally, due to its positive impact on the photooxidation of proteins, vitamin D potentially exhibits a preventive effect in the development of cataract [17, 28, 29]. Moreover, vitamin D is closely related to calcium, and the protective effect of calcium dobesilate against steroid-mediated cataract was reported in animal experiments by Velpandian and colleagues, probably due to its antioxidant and aldose reductase inhibitor properties [30]. Conversely, some studies reported that overloading of intracellular calcium concentrations may cause abnormal degradation of proteins and cell death, which in turn may lead to structural abnormalities in the lens [31, 32].

In the subgroup analysis of sex, we observed a significant association between serum vitamin D level and cataract in female and male. This result is different from Jee’s study, which vitamin D level is associated with cataract in men but not in women [17], possibly because when conducting the subgroup analysis, Jee’s study was not included due to inconsistent numbers of male and female between two tables in their study. Therefore, only two studies were included in our study for the subgroup analysis of sex [16, 24]. Also, Rao’s study reported that serum vitamin D level was protective against nuclear cataract in women younger than 70 years old only [19]. The reason for this discrepancy is unclear, and further studies are warranted.

There are three main types of aged-related cataract: nuclear, cortical, and posterior subcapsular [33]. When considering the different types of cataract, our findings suggest that the relationship between vitamin D level and cataract may vary depending on the types of cataract. Specifically, we observed that vitamin D level was negatively associated with the risk of nuclear cataract and posterior subcapsular cataract, whereas there was no significant association with cortical cataract. The associations between different types of cataract and vitamin D level remain uncertain with varying results across studies. Aidenloo reported that serum vitamin D level was inversely associated with nuclear and cortical cataract but not posterior subcapsular cataract in women [16], while inverse association between serum vitamin D and posterior subcapsular cataract was observed in Bozkurt and Atalay’s studies [34, 35]. Park’s results showed that there was negative association between serum vitamin D and nuclear cataract [18]. Contrarily, there was no significant association between serum vitamin D and nuclear cataract in Rao’s study [19]. These subtype-specific differences may be attributed to variations in the underlying pathophysiological processes involved in the development of different cataract subtypes.

Vitamin D supplement and cataract have been previously investigated. The Beaver Dam Eye Study indicated that there was a protective relationship between vitamin D intake and nuclear cataract [36]. However, other studies revealed that no significant association between vitamin D supplement and cataract [37, 38]. In a recent randomized controlled trial, participants were randomly assigned to receive regular high-dose vitamin D supplement (60,000 IU once per month) or placebo, and were followed up for a median duration of approximately 5 years. The results showed no significant difference in cataract surgery rates between the experimental group and the placebo group [39]. We found that high-level serum Vitamin D reduced the risk of cataract, indicating a potential role of vitamin D supplementation in ophthalmologic practice. Based on the current available evidence, the relationship between vitamin D supplement and cataract remains inconclusive. Further studies of vitamin D supplementation against cataract are warranted.

There are several limitations to this meta-analysis. Firstly, only four studies were included for analysis and the risk of bias in the four articles were rated as having some concerns based on the quality. Additionally, the heterogeneity is high, we conducted subgroup analysis only in sex and different types of cataract. The main cause of heterogeneity is sex. Meanwhile, we conducted the sensitivity analysis which revealed the Jee’s study influenced the meta-analysis results. The reason for this result could be that the study in question had a different study design and measurement method for vitamin D compared to the other three studies. Furthermore, this study included a much larger sample size than the other three studies and took a dominant role in our analysis. The details showed in Table 1. Additionally, various studies have indicated that Vitamin D plays a role in the pathogenesis and progression of diabetes [40]. Vitamin D deficiency increase the risk of developing type 2 diabetes. [41]Meanwhile, diabetes is the most common risk factor for cataract. [42]Among the four studies, two involve data on diabetes mellitus. In Jee’s study [17], the proportion of patients with diabetes was significantly higher in the cataract group compared to the non-cataract group. In the study by Aidenloo [16], the proportion of patients with diabetes were only significantly higher in the PSC group compared to the control group, and no significant differences in serum calcium levels were observed between the different cataract subgroups and the control group. However, since the vitamin D levels of the diabetic patients were not provided, we are unable to perform any further analysis and understanding of potential correlations.

## Conclusion

In this meta-analysis, we reported that there was a possible association between serum vitamin D level and cataract, and indicated that the association between serum vitamin D level and cataract varied across sex and different types of cataract. However, drawing conclusions at this stage seems premature due to the limited number of studies available so far. Therefore, prospective, multicenter, and larger-scale studies are needed in the future.

## Supplementary Information

Below is the link to the electronic supplementary material.

Supplementary file1 (DOCX 43 KB)
